# Impact of renal replacement therapies on olfactory ability: results of a cross-sectional case control study

**DOI:** 10.1007/s40620-021-00983-6

**Published:** 2021-02-24

**Authors:** Valentina Iacono, Gianmarco Lombardi, Giancarlo Ottaviano, Giovanni Gambaro, Gianluigi Zaza

**Affiliations:** 1 Department of Medicine, Renal Unit, University Hospital of Verona, Piazzale A. Stefani 1, 37126 Verona (VR), Italy; 2 Department of Neurosciences, Otolaryngology Section, University of Padova, Padova, Italy

**Keywords:** Olfactory function, Chronic kidney disease, Hemodialysis, Peritoneal dialysis, Kidney transplantation

## Abstract

**Introduction:**

Several studies have suggested that chronic kidney disease (CKD) may be associated with olfactory impairment. However, to date, the impact of renal replacement therapies has only been partly defined.

**Methods:**

We tested the olfactory function of 235 participants [50 kidney transplant recipients (KT), 49 hemodialyzed patients (HD), 30 peritoneal dialysis patients (PD), 51 patients with CKD not on dialysis (ND-CKD) and 55 healthy subjects (HS)] by the Sniffin’ Sticks test (Burghardt®, Wedel, Germany), including the sub-tests for the determination of odor threshold (*T*), odor discrimination (*D*), odor identification (*I*). Each subtest result was then summed up to a composite score, known as the TDI score. The Sino-Nasal Outcome Test-22 (SNOT22), Montreal Cognitive Assessment (MoCA) test and olfactory function Visual Analogue Scale (ofVAS) were also performed.

**Results:**

The mean *TDI* score was significantly lower (and consistent with hyposmia), in HD, PD and ND-CKD compared to HS and KT (ANOVA p < 0.001). Similar results were observed in the *I* and *D* tests, and with the T score, though with regard to the latter, only in PD and ND-CKD patients. Multiple comparisons among groups demonstrated no significant differences between KT and HS. After adjustments for confounding factors, a significant linear association was found between both urea (β − 0.03, p < 0.003) and eGFR (β 0.08, p < 0.001) with TDI score. No significant association was observed between the *TDI* score and the ofVAS score (p = 0.293).

**Conclusions:**

Olfactory impairment affects a large number of CKD patients in the pre-dialysis phase as well as those on dialysis. Kidney transplantation may reverse this condition with a possible positive impact on the quality of life and social behaviors/relationships.

**Graphic abstract:**

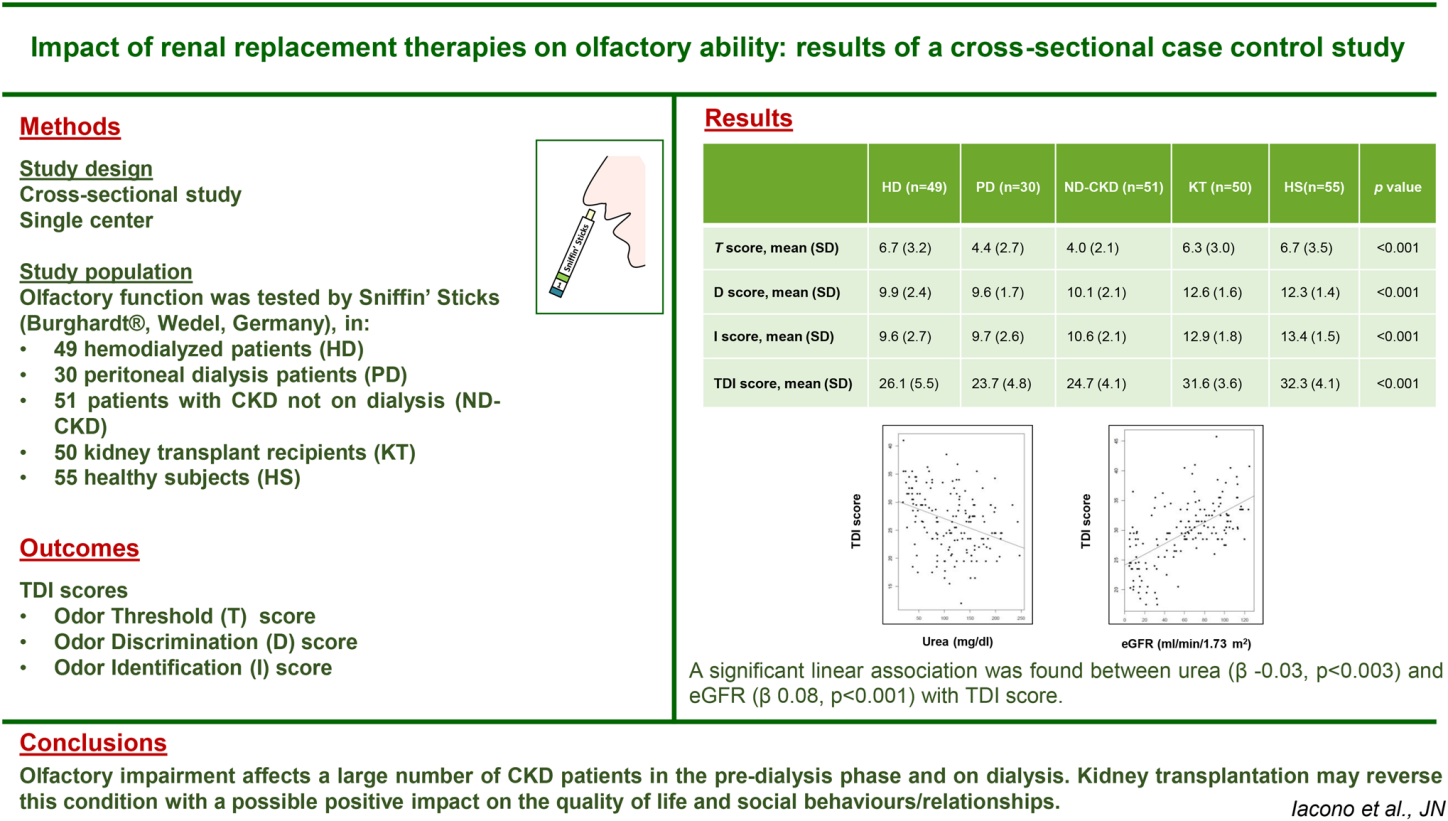

**Supplementary Information:**

The online version contains supplementary material available at 10.1007/s40620-021-00983-6.

## Introduction

Chronic kidney disease (CKD) is a syndrome defined as persistent alterations in kidney structure, function or both, with implications for the health of the individual [1, 2].

Adoption of a healthy diet and pharmacological therapies are required in the early stages to slow down the progression of kidney disease, to reduce morbidity and to improve quality of life [3]. In CKD stage 5 (end stage renal disease, (ESRD)) renal replacement therapies (RRTs), hemodialysis (HD), peritoneal dialysis (PD) or kidney transplantation are necessary to ensure the patient’s survival. Due to the shortage of available donors, the first treatment option for most ESRD patients is either HD or PD.

However, both dialysis procedures are associated with numerous morbidities including central and peripheral neurological complications [4]. Among these, olfactory dysfunction has been reported in more than one study [5–10] although the methods used for the olfactory evaluation were very different and in some cases not universally validated, and therefore the results were frequently conflicting [11, 12].

As reported by Griep et al., renal transplantation can reverse olfactory dysfunction [5]. However, no other studies have addressed this topic.

The reduced perception of odor in CKD patients may negatively impact interpersonal functioning [13]. Specifically, since olfaction is critical for normative social behavior [14] and social relationships are critical for health and reduced mortality [15], olfactory dysfunction may impair interpersonal functioning, thereby increasing mortality risk. In addition, it may contribute to malnutrition and loss of appetite through the decreased smell and perturbed taste sensitivity that characterize CKD [16].

Understanding the link between olfactory impairment and renal disease could play an important role in developing new therapeutic interventions and thus improving clinical outcomes, nutritional status and quality of life in this patient population.

Although some studies have analyzed the relationship between odor perception and CKD, none of them have extensively evaluated odor identification, threshold and discrimination across all types of RRTs, including kidney transplantation. Therefore, the aim of our study was to fully describe the relationship between renal disease and odor impairment and to analyze the impact of RRTs on olfactory function in CKD patients.

## Methods

### Study setting and participants

A total of 235 adult (more than 18 years old) participants [50 kidney transplant recipients (KT), 49 HD patients, 30 PD patients, 51 patients with CKD not on dialysis (ND-CKD) and 55 healthy subjects (HS)] were enrolled (Supplementary Fig. 1). All patients provided written informed consent before participating in the study.

Participants were enrolled between February 2019 and February 2020. All patients were recruited from the out-clinic follow-up service, peritoneal dialysis center and hemodialysis center of the Renal Unit, Department of Medicine, of the University Hospital of Verona (Italy). Healthy subjects (controls) were recruited from the nursing and medical staff of our institution.

Demographic, clinical, and therapeutic data were collected for all participants. A 10 ml blood sample was collected to dose serum creatinine and urea. Renal function was estimated by glomerular filtration rate (eGFR) using the CKD-EPI equation [17]. As surrogate uremic markers, intact parathyroid hormone (iPTH), serum phosphorus (P) and hemoglobin (Hb) were included in the analysis.

At the time of enrollment, the Sino-Nasal Outcome Test-22 (SNOT22) [18] and the Montreal Cognitive Assessment (MoCA) [19] were carried out for each participant. The former is a health-related quality of life (QOL) assessment tool able to assess the degree and effect of rhinosinusitis on health status. It contains 22 items divided into two categories: 10 questions about physical symptoms and 10 questions about QOL, which cover sleep function and psychological issues. Each item is scored from 0 to 5. The sum of each item results in a maximum score of 110. While high scores indicate poor outcome [20, 21], it has been proposed that a score of 0 or 1 for each question is representative of normal olfactory function [22].

MoCA is a cognitive screening tool, which assesses cognitive impairment in different domains: visual–constructional skills, executive functions, attention and concentration, memory, language, conceptual thinking, calculations and spatial orientation. The MoCA is a reliable and valid screening tool for cognitive status in different populations and has been used in many studies in correlation with the olfactory status [23, 24].

For our analysis and data calculation, we included only adult patients (18 years or older) with a normal sense of smell from a self-assessment test (SNOT22 total score less than 22 and with a maximum score of 1 for each question) [25, 26]. Participants with a MoCA score below 25 (possible cognitive impairment), as well as those with a history of smoking, use of beta-blockers or estroprogestinics, previous nasal surgery, malignancies or severe liver disease, asthma or allergies, congestive heart failure or Parkinson’s disease were excluded from the study.

Our cross-sectional, case–control, single center study was approved by the Local Ethics Committee (AOUI Verona, 2025 CESC).

### Assessment of olfactory function

Before undergoing quantitative smell test evaluation, all subjects were asked to self-report their own olfactory function as good, reduced, or no sense of smell [olfactory function Visual Analogue Scale (ofVAS)].

The sniffin’ sticks test (Burghardt®, Wedel, Germany) [27–29] consists of felt pens, the tips of which are impregnated with odorant fluid or odorant substance. The test comprises 3 subtests resulting in 3 scores: odor threshold (*T*), odor discrimination (*D*), and odor identification (*I*) [30]. The smell test sequence was *T*, *D*, and finally *I*. Each subtest result was then summed up to a composite score, known as the TDI score. Subjects with a *TDI* score > 30 were considered to have normal olfactory function (normosmia), subjects with a *TDI* score of 15–30 were considered to have decreased olfactory function (hyposmia), while subjects with a *TDI* score < 15 were considered to have loss of olfactory function (anosmia) [12].

### Outcome and exposures

*TDI* scores were the main outcomes of interest. The *T*, *D*, and *I* scores were analyzed as secondary outcomes. Odor impairment (defined as a *TDI* score less than 30) was ultimately evaluated as exposure in binomial regression.

Covariates assessed for confounding control in regression modeling included age, sex and BMI.

In order to evaluate the relationship between dialysis adequacy and olfactory function we collected all Kt/V [31] dosed during the previous year in HD participants however, only patients with at least 4 Kt/V measurements (every three months) were considered eligible for the analysis. One-year mean Kt/V was used as exposure in regression modeling with TDI score.

### Statistical analysis

Categorical variables were expressed as numbers and percentages. Continuous variables were expressed as means with standard deviations (SDs) (normal distribution) or medians with interquartile ranges (IQRs) (skewed distribution). Normality of distributions were evaluated by visual inspection of histogram, Q-Q plot and Shapiro-Wilkinson test.

Baseline measurements were compared using one-way ANOVA (normally distributed variables), Kruskall Wallis test (skewed distributed variables) and chi-squared test for categorical variables.

Olfactory function assessments among groups were compared using ANOVA. Comparisons among groups were then tested using Tukey studentized range adjustment with sandwich estimator.

As sensitivity analysis, the independent relationship between the RRT group and odor impairment (analyzed as a categorical binomial variable) was evaluated using multivariable logistic regression modeling.

Linear regression models were performed to assess the relationship between renal function (eGFR, urea) and *TDI* score, after which, iPTH, P and Hb were also evaluated in regression modeling for association with *TDI*. A Poisson regression model was ultimately built to evaluate the association between *TDI* score and ofVAS score for each participant.

As secondary analysis the association between dialysis adequacy and olfactory function was evaluated using one-year mean Kt/V as independent variable in a linear regression model with *TDI* score.

All data were analyzed with R version 3.4.4 (R Foundation for Statistical Computing Platform). A p value < 0.05 was considered statistically significant.

## Results

### Study population

Overall, 235 participants were included in the study. The study population was stratified into the following groups; healthy subjects with normal kidney function (*n* = 55), ND-CKD patients (*n* = 51), PD patients (*n* = 30), HD patients (*n* = 49), KT patients (*n* = 50).

Baseline characteristics of the study population are reported in Table 1.

**Table 1:** Baseline characteristics of the study population

	Overall	Healthy subjects	CKD patients	*p* value
*n*	235	55	180	0.014
Age, mean (SD), year	58.0 (14.5)	53.9 (13.5)	59.3 (14.6)	0.001
Males, *n* (%)	146 (62.1)	23 (41.8)	123 (68.3)	0.594
BMI, mean (SD), Kg/m^2^	25.9 (4.9)	26.2 (5.1)	25.8 (4.9)	0.001
Diabetes, *n* (%)	34 (14.5)	0 (0.0)	34 (18.9)	0.001
SNOT22 test, median (IQR)	10.0 (4.0, 15.0)	13.0 (4.5, 20.0)	9.0 (4.0, 13.0)	0.101
MoCA score, median (IQR)	27.0 (26.0, 29.0)	28.0 (27.0, 29.0)	27.0 (26.0, 29.0)	0.883
ofVAS, median (IQR)	8.0 (7.0, 9.0)	8.0 (7.0, 9.0)	8.0 (7.0, 9.0)	

*SNOT22* Sino-Nasal Outcome Test, *MoCA* Montreal Cognitive Assessment, *ofVAS* olfactory function Visual Analog Scale

All CKD participants (including all patients in the pre-dialysis phase and those already on dialysis) were older than the healthy subjects, and there was a higher percentage of males. No significant differences were observed for ofVAS score or MoCA score between the two groups (Table 1).

Interestingly, when data were stratified by renal replacement therapy (Table 2), HD, PD and ND-CKD patients were older compared to KT. As expected, higher levels of urea were observed in HD, PD and ND-CKD patients compared to KT patients.

**Table 2:** Baseline characteristics of the study population stratified by RRT

	HD patients	PD patients	ND-CKD patients	KT patients	*p* value
*n*	49	30	51	50	
Age, mean (SD), year	59.6 (13.3)	66.2 (14.9)	58.0 (15.0)	56.18 (14.2)	0.023
Males, *n* (%)	31 (63.3)	23 (76.7)	39 (76.5)	30 (60.0)	0.194
BMI, mean (SD), Kg/m^2^	28.1 (5.5)	24.4 (4.6)	26.2 (4.6)	23.96 (3.74)	< 0.001
Diabetes, *n* (%)	19 (38.8)	2 (6.7)	5 (9.8)	8 (16.0)	< 0.001
Nephropathy, *n* (%)					0.003
ADPKD	6 (12.2)	5 (16.7)	8 (15.7)	11 (22.0)	
CAKUT	4 (8.2)	3 (10.0)	4 (7.8)	2 (4.0)	
CKDu	11 (22.4)	13 (43.3)	19 (37.3)	15 (30.0)	
Diabetic nephropathy	13 (26.5)	1 (3.3)	3 (5.9)	1 (2.0)	
Glomerulonephritis	8 (16.3)	5 (16.7)	10 (19.6)	17 (34.0)	
Others	2 (4.1)	1 (3.3)	7 (13.7)	3 (6.0)	
Urinary obstruction	1 (2.0)	0 (0.0)	0 (0.0)	1 (2.0)	
Vascular disease	4 (8.2)	2 (6.7)	0 (0.0)	0 (0.0)	
Vintage RRT, median (IQR), year	2.0 (1.5, 4.0)	2.0 (0.7, 3.0)	–	7.0 (2.3, 12.8)	< 0.001
eGFR, median (IQR), mL/min/1.73 m^2^*	–	–	14.1 (8.7, 31.0)	64.3 (47.0, 80.5)	< 0.001
Urea, mean (SD), mg/dL	154.1 (37.1)	131.8 (26.0)	111.1 (58.4)	54.0 (26.1)	< 0.001
SNOT22 test, median (IQR)	8.0 (4.0, 11.0)	6.0 (3.0, 10.8)	11.0 (4.5, 15.5)	8.0 (3.0, 13.8)	0.082
MoCA score, median (IQR)	27.0 (26.0, 28.0)	26.5 (26.0, 27.0)	27.0 (27.0, 29.0)	28.0 (26.3, 29.0)	0.031
ofVAS, median (IQR)	8.0 (7.0, 10.0)	8.0 (7.0, 9.0)	8.0 (7.0, 9.0)	8.0 (7.0, 9.0)	0.458

*SNOT22* Sino-Nasal Outcome Test, *MoCA* Montreal Cognitive Assessment, *ofVAS* olfactory function Visual Analog Scale, *ADPKD* Autosomal Dominant Polycystic Kidney Disease, *CAKUT* Congenital Anomalies of Kidney and Urinary Tract, *CKDu* Chronic Kidney Disease of unknown origin, *HD* hemodialysis, *PD* peritoneal dialysis, *ND-CKD* chronic kidney disease not on dialysis, *KT* kidney transplantation

*Estimated GFR CKD EPI

### Olfactory impairment in renal replacement therapies

*T* score, *I* score, *D* score and comprehensive *TDI* score were assessed for each group. As reported in Table 3 the mean *TDI* score was significantly lower in HD and ND-CKD patients compared to healthy subjects and KT (ANOVA p < 0.001).

**Table 3:** Olfactory assessment in the study population

	HD patients	PD patients	ND-CKD patients	KT patients	Healthy subjects	*p* value
Odor threshold (*T*) score, mean (SD)	6.7 (3.2)	4.4 (2.7)	4.0 (2.1)	6.3 (3.0)	6.7 (3.5)	< 0.001
Odor discrimination (*D*) score, mean (SD)	9.9 (2.4)	9.6 (1.7)	10.1 (2.1)	12.6 (1.6)	12.3 (1.4)	< 0.001
Odor identification (I) score, mean (SD)	9.6 (2.7)	9.7 (2.6)	10.6 (2.1)	12.9 (1.8)	13.4 (1.5)	< 0.001
*TDI* score, mean (SD)*	26.1 (5.5)	23.7 (4.8)	24.7 (4.1)	31.6 (3.6)	32.3 (4.1)	< 0.001

*HD* hemodialysis, *PD* peritoneal dialysis, *ND-CKD* chronic kidney disease not on dialysis, *KT* kidney transplantation, *TDI score* (OT-score plus OD-score plus OI-score)

*Normosmia is defined as a TDI score > 30

**Fig. 1:**
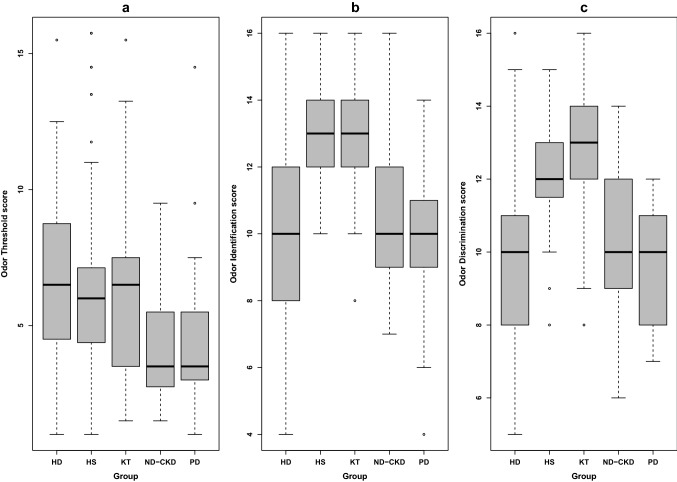
Box plots of olfactory function tests. **a** Odor Threshold score, **b** Odor Identification score, **c** Odor Discrimination score for each group

Multiple inter-group comparisons demonstrated no significant differences between KT and HS. On the other hand, significant differences with lower *I* and *D* scores were observed in PD, HD and ND-CKD patients compared with the HS and KT groups. A similar trend was also found in *T* scores only in PD and ND-CKD participants (Supplementary Table 1, Fig. 1). Overall, 130 out of 180 CKD participants (72.2%) showed olfactory impairment. Multivariable adjusted logistic modeling demonstrated the independent relationship of PD, HD, and ND-CKD patients with odor impairment (Table 4).

**Table 4:** Odor impairment and CKD

	HD patients	PD patients	ND-CKD patients	KT patients	Healthy subjects
Odor impairment, *n* (%)	37 (75.5)	28 (93.3)	48 (94.1)	17 (34.0)	16 (29.1)
Log OR (95% CI)	2.02 (1.17, 2.92) *p* < 0.001	3.53 (2.18, 5.43) *p* < 0.001	3.66 (2.49, 5.18) *p* < 0.001	0.23 (-0.60, 1.06) *p* 0.589	1.00 (Reference)
Log OR (95% CI)*	1.98 (1.04, 2.97) *p* < 0.001	3.41 (1.92, 5.41) *p* < 0.001	4.04 (2.73, 5.69) *p* < 0.001	0.16 (-0.76, 1.09) *p* 0.728	1.00 (Reference)

*HD *hemodialysis, *PD *peritoneal dialysis, *ND-CKD* chronic kidney disease not on dialysis, *KT* kidney transplantation

^*^ Adjusted for: Age, Sex, BMI

No significant association was observed between *TDI* score and ofVAS score (Poisson regression, *p* = 0.293).

### Odor perception and indicator of renal dysfunction

**Fig. 2:**
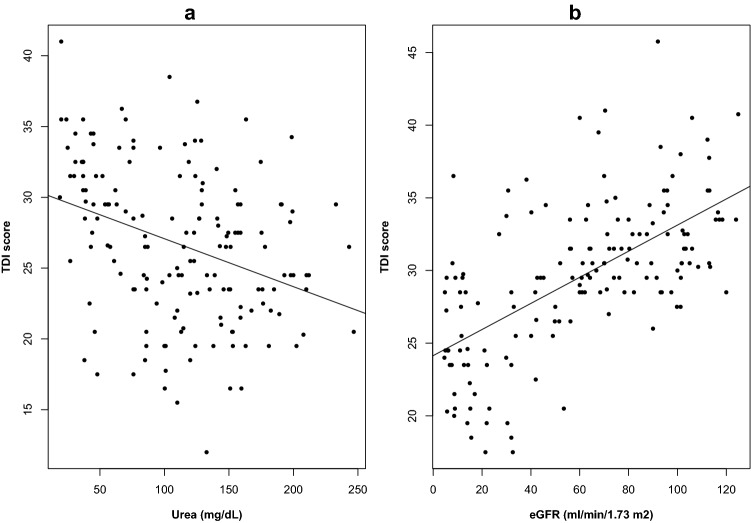
Relationship between markers of renal function and olfactory impairment. **a** Distribution of Urea (mg/dL) and TDI score in the study population including healthy subjects (*r* = − 0.35, p < 0.001). **b** Distribution of eGFR (ml/min/1.73 m^2^) and TDI score for patients not on dialysis including healthy subjects (*r* = 0.61, p < 0.001)

A significant linear association was found between *TDI* score and urea (negative correlation, β − 0.03, *p* < 0.001) (Fig. 2a), as well as between *TDI* score and eGFR (positive correlation, β 0.09, *p* < 0.001) (Fig. 2b). The independent relationship was confirmed even after adjustment for potential confounders, age and BMI (urea, negative correlation, β − 0.03, *p* < 0.001*;* eGFR positive correlation, β 0.08, *p* < 0.001).

As secondary analysis, we found a significant negative relationship between iPTH levels and TDI [β coefficient − 0.03 (SE 0.02), p = 0.037] and a significant positive relationship between Hb levels and TDI [β coefficient 0.77 (SE 0.25), p = 0.002]; no significant association was observed between phosphorus levels and TDI [β coefficient − 0.15 (SE 0.15), p = 0.325]. However, after introducing urea levels into the same models (multivariable linear regression) the significant effect of iPTH or Hb completely disappeared, suggesting that urea fully mediates such association.

### Olfactory function and hemodialysis

**Fig. 3:**
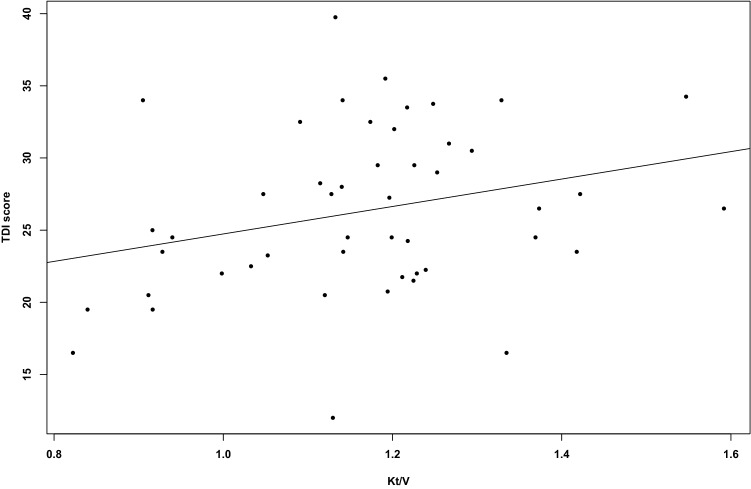
Relationship between dialysis adequacy and olfactory function. Correlation between Kt/V (one-year mean Kt/V) and TDI score in HD patients (*r* = 0.28, p = 0.054)

To evaluate the contribution of dialysis adequacy on the olfactory function we collected the Kt/V measured for each patient one year before the start of our study. We observed that higher mean Kt/V was associated with increased *TDI* score (β 0.01, *p =* 0.054) (Fig. 3).

## Discussion

In this study olfactory function of CKD patients in the pre-dialysis phase, those undergoing HD or PD, and KT patients together with healthy controls have been assessed using validated olfactory tests [32]. Our results confirm, in a large and well-phenotyped patient cohort, previous findings demonstrating the association between chronic renal impairment and olfactory ability [5–9, 13].

CKD is a global health burden that affects more than 10% of the population [33] worldwide to some degree. Together with cardiovascular diseases, neurological disorders may also have a negative clinical impact on this large patient population [34]. In particular, olfactory deficits may affect CKD patients by altering the flavor of food which might thus result in food aversion, and anorexia [35, 36]. These symptoms can then contribute to malnutrition [37, 38].

Few reports have addressed olfactory impairment in CKD and the impact of the various RRT modalities. Only one study [5], involving a smaller cohort of patients (n = 28), that was published more than 20 years ago, reported recovery of olfactory function after kidney transplantation.

Our analysis showed that approximately 70% of our CKD patients have reduced olfactory perception, especially in terms of identification and discrimination, and that this condition could be related to possible neurological disorders. There may be many reasons for the association between renal impairment and the development of olfactory dysfunction including uremic neuropathy, a condition characterized by olfactory peripheral epithelial neuron alterations and central processing dysfunctions following accumulation of uremic toxins.

In particular, the neurons of the olfactory epithelium are constantly regenerated for their life-span, and any toxin or agent that can slow-down or interrupt their cell growth could dramatically influence their functions [39, 40].

Moreover, the high prevalence of diabetes in the CKD population could play an important role in olfactory dysfunction. Although the pathophysiology has not yet been clearly demonstrated, a relationship between diabetes and olfactory impairment has been suggested [41]. In our study population we observed an increased prevalence of olfactory impairment in diabetic participants (17.8% in participants with odor impairment vs 9.0% in participants with normal olfactory function), albeit it did not reach the statistical significance  (p 0.094).

Olfactory impairment in these patients may be reversible as reported by Griep and coworkers in KT patients, but also after dialysis  [11]. In this regard, the latter authors proposed that adequate clearance of uremic toxins seemed to improve olfactory identification. This is in line with our results demonstrating that there is a linear positive correlation between hemodialysis urea clearance (one-year mean Kt/V) and olfactory function (TDI score). However, the threshold test was impaired only in the ND-CKD and PD groups; HD patients did not show a reduction in the *n*-butanol threshold function. A similar result was observed by Landis and co-workers who did not find significant n-butanol threshold modifications after dialysis in their patients. Similarly, Frasnelli et al., and Vreman et al., did not find significant differences in odor threshold scores between CKD patients and controls [7, 10]. It is therefore possible that CKD may disproportionately affect peripheral odor sensitivity [11].

In our opinion, an important result of our study is that no significant differences were observed between KT recipients and healthy subjects. This interesting result likely reflects the ability of transplantation to restore both renal and olfactory function, and it clearly demonstrates how immunosuppressive therapy, although administered for several years, does not affect the olfactory system.

Notably, no significant association was found between the sum of *T*, *D*, *I* scores (*TDI* score) and ofVAS, suggesting that most CKD patients are unaware of their disease-associated olfactory decline.

Our study has some limitations. The observational and cross-sectional nature of the study does not allow us to define a causal relationship between urea and olfactory impairment; age and sex differences between cases and controls could not be completely fixed in multivariable regression modeling. We did not evaluate the direct association between the specific type of uremic toxin and odor dysfunction, or the association between markers of malnutrition and inflammation/oxidative stress factors and olfactory function. Finally, due to the unavailability of data we could not explore the role of PD adequacy over time on olfactory impairment. New prospective studies should be performed to address such issues.

However, our study has comprehensively analyzed olfactory function among all types of RRTs by using robust statistics on the largest cohort of CKD patients.

In conclusion, we showed that olfactory impairment is a condition that affects a large number of CKD patients in the pre-dialysis phase as well as those already on dialysis treatment, with a significant impact on the quality of life even if CKD patients are often unaware of being affected by this disorder. Retained uremic toxins are probably the main determinants of such impairment which itself is a marker of neurologic dysfunction in the uremic state. Interestingly, kidney transplantation may reverse this condition.

In the future, larger clinical studies should be conducted in order to evaluate the causal association between specific uremic toxins and odor impairment. Evaluation of olfactory performance could be a potential marker of uremic toxicity and a valid surrogate marker of dialysis adequacy. At the same time, detecting olfactory impairment could become a useful tool for identifying patients at risk of malnutrition. Furthermore, it would be interesting to evaluate the effects of olfactory training [30] in these patients in order to develop novel therapeutic interventions.

## Supplementary Information

Below is the link to the electronic supplementary material.

Supplementary file1 (PDF 31 KB)

Supplementary file2 (PDF 80 KB)
