# Supplementary material for: Impact of renal replacement therapies on olfactory ability: results of a cross-sectional case control study
Source: J Nephrol. 2021 Feb 24;35(1):223–32. doi: 10.1007/s40620-021-00983-6 (PMC8803626; doi:10.1007/s40620-021-00983-6)
Supplement: Supplementary file 1 — Supplementary file1 (PDF 31 KB) [file 40620_2021_983_MOESM1_ESM.pdf]

**Supplementary Table 1. Multiple comparison between groups on olfactory impairment**

| Group comparison | Odor Threshold score* | Odor Identification score* | Odor Discrimination score* | TDI score* |
|---|---|---|---|---|
| **PD vs ND CKD** | 0.965 | 0.452 | 0.797 | 0.875 |
| **HD vs ND CKD** | <0.001 | 0.205 | 0.991 | 0.556 |
| **KT vs ND CKD** | <0.001 | <0.001 | <0.001 | <0.001 |
| **Healthy subjects vs ND CKD** | <0.001 | <0.001 | <0.001 | <0.001 |
| **HD vs PD** | 0.005 | 1.000 | 0.977 | 0.226 |
| **KT vs PD** | 0.025 | <0.001 | <0.001 | <0.001 |
| **Healthy subjects vs PD** | 0.007 | <0.001 | <0.001 | <0.001 |
| **KT vs HD** | 0.956 | <0.001 | <0.001 | <0.001 |
| **Healthy subjects vs HD** | 0.999 | <0.001 | <0.001 | <0.001 |
| **Healthy subjects vs KT** | 0.972 | 0.612 | 0.832 | 0.872 |

* *p* value, Tukey test with sandwich estimator
