# Supplementary figures and images for: Impact of renal replacement therapies on olfactory ability: results of a cross-sectional case control study
Source: J Nephrol. 2021 Feb 24;35(1):223–32. doi: 10.1007/s40620-021-00983-6 (PMC8803626; doi:10.1007/s40620-021-00983-6)

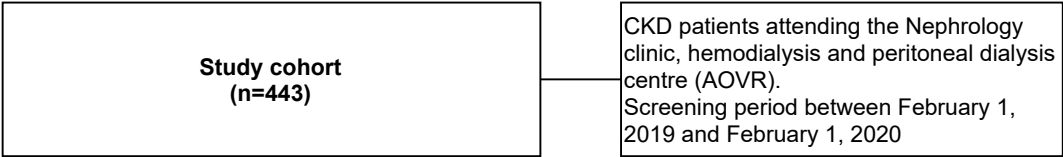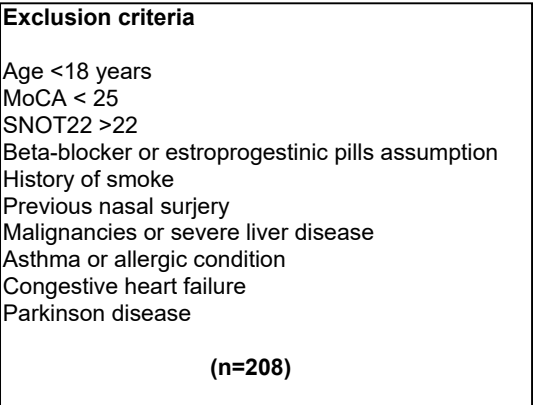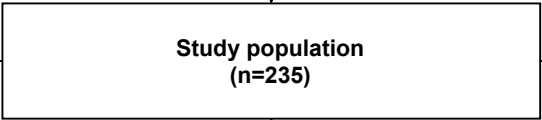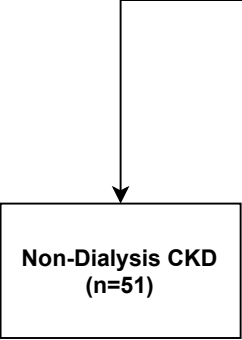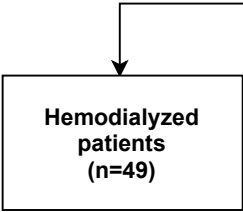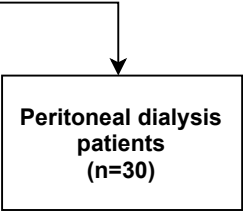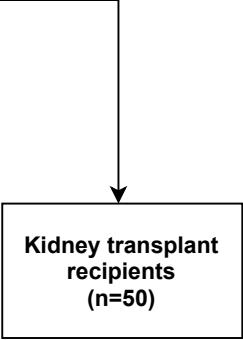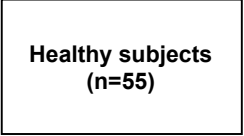

Supplement: Supplementary file 2 — Supplementary file2 (PDF 80 KB) [file 40620_2021_983_MOESM2_ESM.pdf]
